# Bilateral Central Serous Chorioretinopathy Associated With Tuberculosis in a Female Patient

**DOI:** 10.7759/cureus.97557

**Published:** 2025-11-23

**Authors:** William E Holmes, Sriharanathan Poopalaratnam

**Affiliations:** 1 Vitreo-Retinal Surgery, National Hospital Kandy, Kandy, LKA; 2 Medicine, University of Exeter, Exeter, GBR

**Keywords:** anti-tuberculosis therapy, central serous chorioretinopathy, rifampicin, subretinal fluid, tuberculosis (tb)

## Abstract

We present a novel case of central serous chorioretinopathy (CSCR) associated with tuberculosis in a female patient. A 29-year-old female was referred with bilateral atypical CSCR following steroid treatment for suspected optic neuritis. Examination showed bilateral hypermetropic shift and foveal subretinal fluid (SRF). Optical coherence tomography (OCT) confirmed bilateral serous retinal detachment, with associated pigment epithelial detachments in the right eye. A raised erythrocyte sedimentation rate prompted multidisciplinary investigation, eliciting radiological and serological findings consistent with latent pulmonary tuberculosis. Follow-up four days following commencing anti-tuberculosis treatment showed reduction of SRF bilaterally. Complete resolution and return to visual baseline was observed at three months. Prompt CSCR resolution might have been secondary to rifampicin inducing steroid metabolism or effective anti-tuberculosis treatment. Early investigation for tuberculosis in CSCR may enable earlier diagnosis, improving visual outcomes.

## Introduction

Central serous chorioretinopathy (CSCR) is an idiopathic pachychoroid disease, characterised by localised serous detachment of the neurosensory retina from the retinal pigment epithelium (RPE), with perifoveal accumulation of subretinal fluid (SRF) [1-3].

The pathophysiology of CSCR is poorly understood, but choroidal hyperpermeability secondary to venous overload and RPE pump dysfunction is suspected [1,2]. CSCR is most strongly associated with glucocorticoids; endogenous or exogenous, irrespective of dose or duration [4]. Other associations with CSCR include male sex, psychological stress, type A personality, hypertension, hyperandrogenism, sleep apnoea, gastroesophageal reflux and Helicobacter pylori infection [2,5,6].

Patients with CSCR can suffer reduced visual acuity (VA) and central scotoma, with or without metamorphopsia, micropsia, dyschromatopsia or hypermetropic shift. CSCR is typically an acute self-limiting disease over three to six months, with spontaneous resolution of SRF and return to baseline VA in up to 84% of cases [1,2,7]. Steroids should be withdrawn if possible in cases associated with their use [4,7]. Chronic or recurrent disease can precipitate photoreceptor loss, diffuse RPE epitheliopathy, RPE tears, choroidal neovascularization and central macular oedema, with permanent visual sequelae [2,3,8]. Focal laser photocoagulation, and low-dose photodynamic therapy with verteporfin are well-evidenced treatment modalities [2,3,5,8]. Recent evidence suggests systemic mineralocorticoid receptor antagonists (spironolactone or eplerenone), progesterone antagonists (mifepristone), rifampicin, beta-blockers (propranolol), aspirin and carbonic anhydrase inhibitors may induce resolution [1,2,8,9].

Investigations to aid diagnosis include optical coherence tomography (OCT), fluorescein angiography (FA), fundus autofluorescence (FAF) and indocyanine green angiography (ICGA) [2]. Although no universally accepted classification system exists, CSCR is often divided into acute, chronic or recurrent depending on disease duration, course and fundus findings [3,10]. More recently, CSCR has been classified as simple or complex, based on the diameter of RPE lesions on FAF [2,3].

Herein, we report a novel case of bilateral CSCR associated with tuberculosis (TB) in a female patient, with prompt resolution of sub-retinal fluid post anti-tuberculosis treatment.

## Case presentation

A 29-year-old Sri Lankan female was referred to our department for investigation of acute bilateral CSCR for investigation. One month previously she had presented to a general physician with cough and fever, was investigated by a neurologist, and found to have bilateral optic disc swelling. A diagnosis of optic neuritis was suspected, and the patient started on glucocorticoid therapy: three days of intravenous methylprednisolone (1g/day), followed by 11 days of oral prednisolone (1 mg/kg/day) with a short weaning course.

She was referred to the ophthalmology department of a district general hospital shortly following this with a three-day history of painless visual loss. She was systemically well at presentation, with no previous medical, refractive or ophthalmic history of note. On examination, VA was 6/18 bilaterally, with bilateral disc oedema. The patient was referred to our vitreoretinal clinic for investigation.

**Figure 1:**
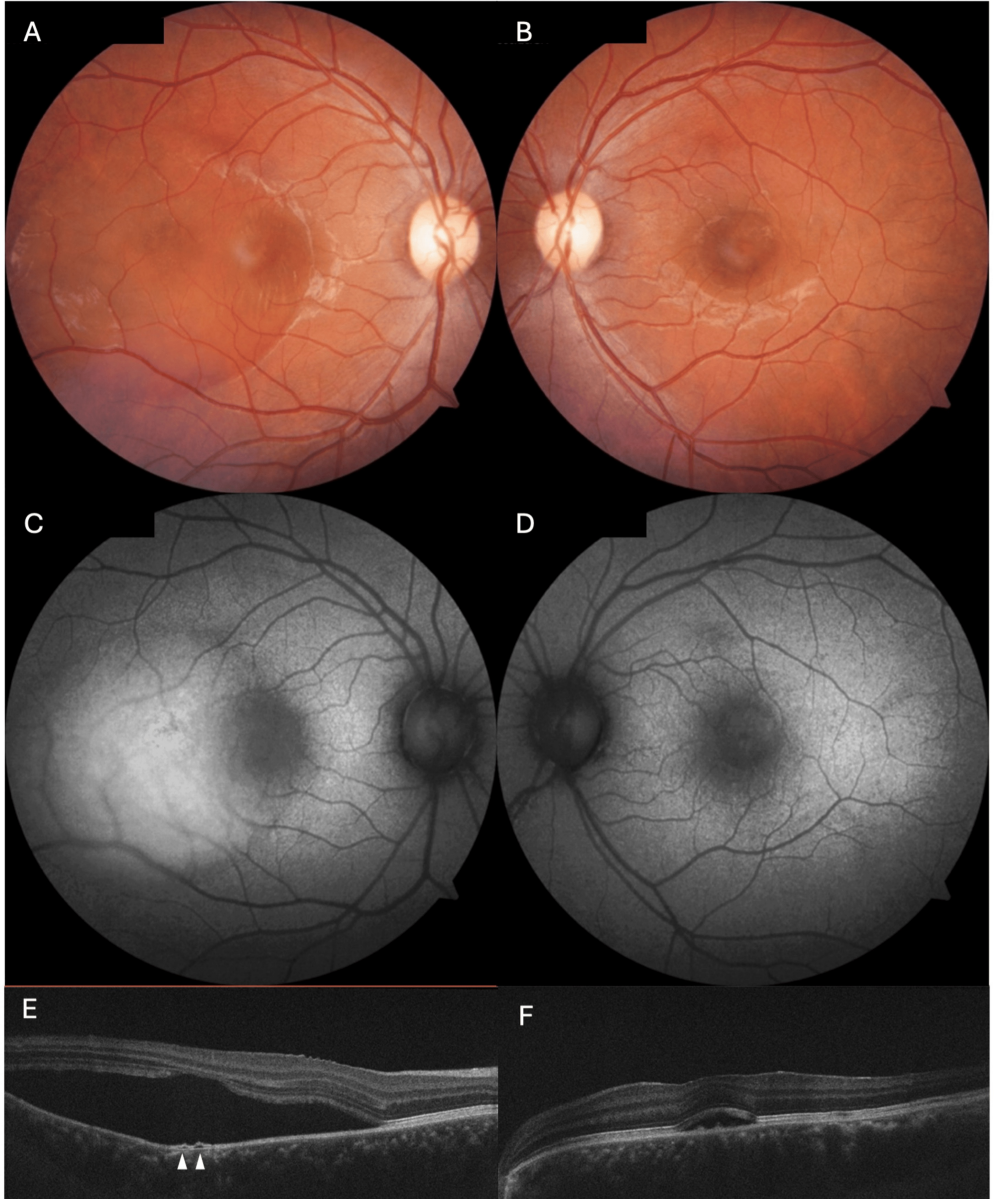
A 29-year-old female presenting with bilateral acute central serous chorioretinopathy (CSCR) (A) Right fundus photo showing significant subretinal fluid (SRF) affecting the macula and extending temporally (B) Left fundus photo showing smaller accumulation of SRF confined to the macula (C) Right fundus autofluorescence (FAF) showing diffuse hyper-autofluorescence within SRF with small region of hypo-autofluorescence (D) Left FAF with macular hypo-autofluorescence (E) Optical coherence tomography (OCT) of right eye showing serous detachment of the neurosensory retina (NSR) from retinal pigment epithelium (RPE), with two small pigment epithelial detachments (PEDs) (arrowheads) (F) OCT left eye showing smaller serous detachment of NSR.

Best corrected visual acuity (BCVA) testing identified bilateral hypermetropic shift: right eye (RE) 0.2 logMAR with +0.75DS, left eye (LE) 0.0 LogMAR with +0.5DS. Anterior segment examination was normal, and fundoscopy showed no signs of intraocular inflammation or disc oedema. However, significant bilateral foveal SRF was identified (Figure 1A-1B). OCT confirmed RE serous neurosensory detachment with a small pigment epithelial detachment (PED) (Figure 1E), and LE foveal SRF (Figure 1F). FAF showed a region of hypo-autofluorescence over the left fovea one disc diameter in size (Figure 1C), and RE hyper-autofluorescence five disc diameters in size with a small region of hypo-autofluorescence (Figure 1D). There were no chronic changes noted on FAF, and the patient declined FA. 

Blood investigation results from the initial presentation with fever showed an elevated erythrocyte sedimentation rate (ESR) of 50mm/hr (Normal range: <20mm/hr) with normal levels of C-reactive protein (CRP), white blood cells, haemoglobin, and platelets. Urinalysis was normal, and antinuclear-antibody negative.

It was suspected that the CSCR was secondary to the exogenous glucocorticoid given for suspected optic neuritis, thus this was immediately withdrawn. However, the persistently elevated ESR with normal CRP prompted further investigation. Referrals to a dermatologist and rheumatologist were made to exclude underlying systemic autoimmune disease contributing to ocular pathology. The rheumatologist noted right apical shadows on chest X-ray, thus referred the patient to a respiratory physician for further evaluation. A Mantoux test was positive (14 mm) and GeneXpert test (Cepheid, Sunnyvale, CA, USA) was weakly positive for Mycobacterium tuberculosis complex. Considered alongside the aforementioned history of fever and cough, a diagnosis of latent pulmonary tuberculosis was made, and the patient started on anti-tuberculosis treatment (ATT): rifampicin, ethambutol, isoniazid, and pyrazinamide.

**Figure 2:**
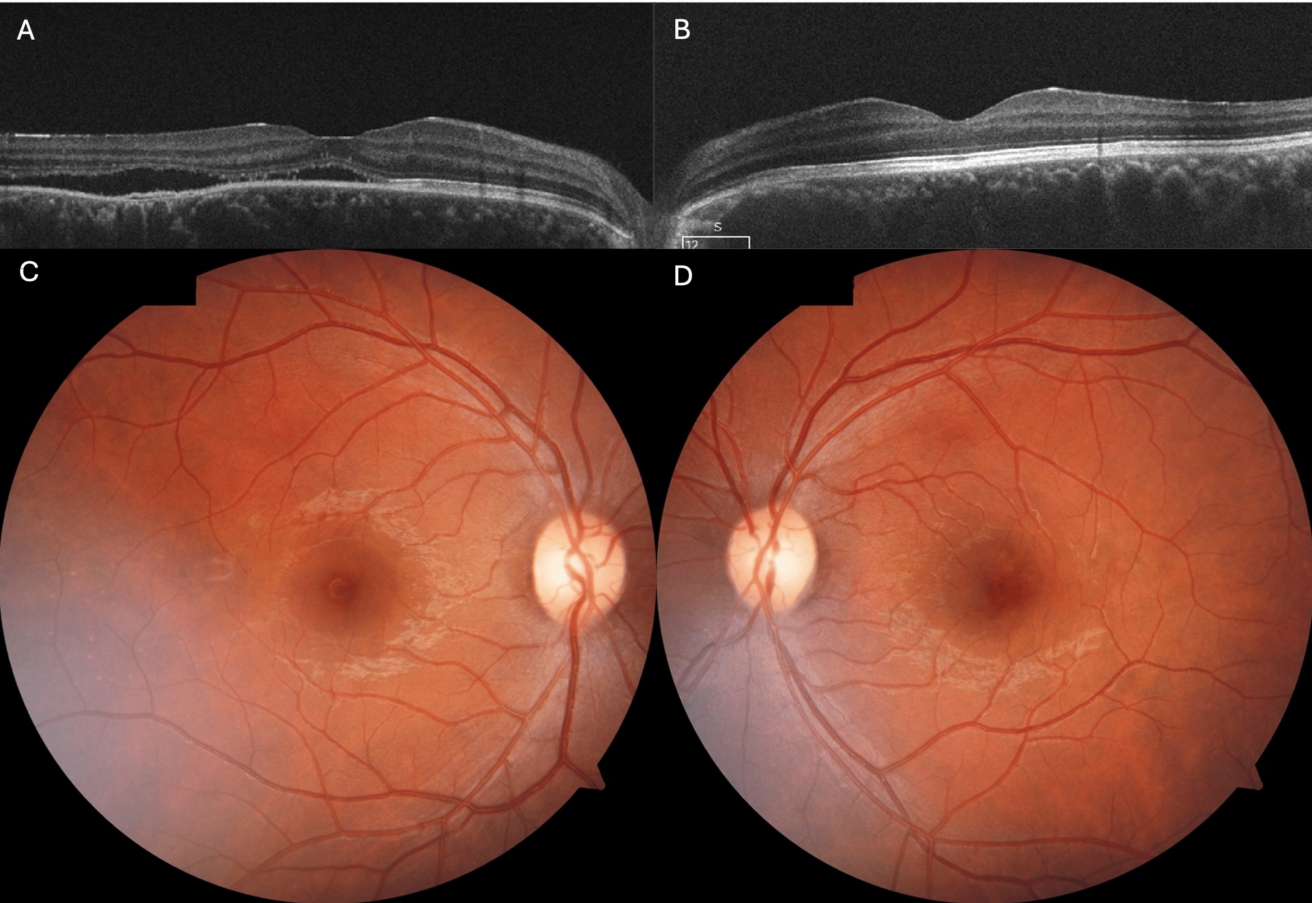
A 29-year-old female presenting with bilateral acute central serous chorioretinopathy (CSCR), following initiation of anti-tuberculosis treatment (ATT) (A) Right eye optical coherence tomography (OCT) four days post commencing ATT showing near complete resolution of subretinal fluid (SRF), with persistence of pigment epithelial detachment (PED). (B) Left eye OCT four days post commencing ATT showing complete resolution of SRF. Right (C) and left (D) fundoscopy at three months follow-up.

At the next ophthalmic follow up 15 days after withdrawing steroids, and four days post commencing ATT, a reduction in hyperopic shift was noted, and OCT scan confirmed a near complete resolution of RE CSCR and complete LE CSCR resolution (Figure 2). Unaided VA was RE 0.3 LogMAR, LE 0.0 LogMAR, with RE BCVA being 0.2 LogMAR with +0.5 DS. Three months later unaided VA was 0.0 LogMAR bilaterally, with near normal fundoscopy and OCT findings. The patient reported no ongoing visual disturbance.

## Discussion

Ocular TB is a rare extrapulmonary manifestation of Mycobacterium tuberculosis (MTB) infection, most commonly arising from secondary haematogenous spread from pulmonary TB rather than primary invasion of ocular tissue [11-13]. A myriad of ocular manifestations exist, owing to the ability of MTB to infect almost all ocular structures [11-13]. Manifestations are typically inflammatory in nature, with choroidal involvement most common, but both granulomatous and non-granulomatous anterior, intermediate, posterior and panuveitis have been reported, as has retinal vasculitis [10-12]. Non-inflammatory retinal involvement is rare; to date, only six cases of CSCR associated with latent TB infection have been reported [5,6,10,13-15]. These cases all occurred in middle-aged males, a population with higher rates of both CSCR and intraocular MTB involvement [2,6,11]. The occurrence of idiopathic CSCR in a female patient is relatively uncommon in comparison, and a case associated with TB in a female patient has not previously been reported in the literature. Thus, a level of caution is required before assuming simultaneous occurrence is correlated, particularly in endemic populations. Previous case reports have stated similar reservations regarding any potential causative association between MTB and CSCR, versus coincidence [12,13]. Our case report has further reservations due to a history significant for methylprednisolone administration; steroid-induced CSCR has no male predominance, typically presents bilaterally, and is frequently associated with PEDs, all features of our case [4]. Despite this, our patient’s rapid response to ATT is of interest, since withdrawal of steroids alone would typically not explain the prompt resolution of CSCR seen in our case [16].

Rifampicin, a bactericidal anti-TB drug, is a cytochrome P4503A4 inducer, increasing hepatic metabolism of circulating endogenous steroids [6,8,9,13,14,17]. Isolated case reports and retrospective case series regarding its usage in acute CSCR have reported rapid resolution of SRF and return to baseline VA [9,14]. Larger prospective interventional studies support this assertion; however, much debate remains regarding optimal dosage and duration of rifampicin therapy, with respect to maximising visual outcomes and minimizing incidence of adverse effects [7,8,18]. Despite this, the general consensus is that rifampicin offers a potential cost-effective and readily available treatment for CSCR, with our case offering further anecdotal evidence. However, concurrent use of other ATT drugs alongside rifampicin, in a case complicated by TB infection, makes it impossible to ascertain whether resolution of SRF was secondary to rifampicin-induced steroid metabolism, or the anti-microbial action of an effective ATT regimen.

SRF analysis in patients with TB has identified MTB DNA, with authors postulating apoptotic and inflammatory processes as possible aetiological mechanisms implicated in inducing vascular hyperpermeability and consequent CSCR [2,6,10,19]. However, any underlying inflammatory mechanism is tenuous due to the strong association between CSCR and glucocorticoids, which are by nature anti-inflammatory [1,2]. Previous cases’ reports of persistent disease despite use of traditional treatment modalities, with rapid resolution of CSCR post ATT, add further plausibility to microbial-based pathological mechanism [6,10].

Anecdotally, rifampicin monotherapy has been reported to be effective in maintaining remission following an initial course of ATT [14]. However, in another report, monotherapy without preceding ATT had little therapeutic effect and resulted in disease recurrence, suggesting initial eradication of MTB infection is required to achieve CSCR resolution in cases associated with TB [6]. Failure to eradicate MTB infection may result in recurrent disease, with higher incidence of adverse visual outcomes and failure to return to visual baseline. In endemic regions, clinicians should maintain a low threshold for investigating underlying TB in cases of CSCR, even in patients without pulmonary symptoms, to enable early diagnosis to minimise adverse visual outcomes [10]. The cause-association dilemma remains a significant problem with respect to case reports such as ours, highlighting the need for further studies to investigate possible roles of MTB in CSCR pathogenesis.

Unfortunately, it is commonplace in our department to observe adverse ocular side effects following initiation of glucocorticoids for presumed optic neuritis (ON). We reiterate the need for multidisciplinary collaboration in the diagnosis and management of patients with neuro-ophthalmic conditions. Despite their role in the pathogenesis of CSCR not being fully elucidated, the increased risk associated with systemic glucocorticoids cannot be understated [4,8]. Interesting treatment predicaments arise when CSCR occurs in association with ON; whether to stop or continue steroids requires both careful case-by-case deliberation by the clinician and informed patient consent [4].

## Conclusions

An infectious aetiology may be implicated in CSCR associated with tuberculosis infection. The potential for TB to precipitate recurrent disease results in an increased risk of adverse visual outcomes. Thus, we reiterate the need for a low threshold for TB investigation in endemic regions, allowing for timely investigation, diagnosis, and management in order to preserve visual function. Resolution of subretinal fluid in cases of CSCR associated with MTB infection may result from rifampicin’s anti-glucocorticoid effect, or alternatively the anti-microbial action of an anti-tubercular regimen.
